# Masseter and temporalis muscle electromyography findings after lower third molar extraction

**DOI:** 10.4317/medoral.21992

**Published:** 2017-12-24

**Authors:** José-María Buesa-Bárez, María Martín-Ares, Natalia Martínez-Rodríguez, Cristina Barona-Dorado, Javier Sanz-Alonso, Jorge Cortés-Bretón-Brinkmann, José-María Martínez-González

**Affiliations:** 1 Lecturer in Oral Surgery, Faculty of Dentistry, Complutense University of Madrid, Spain; 2 Associates lecturer in Oral Surgery, Faculty of Dentistry, Complutense University of Madrid, Spain; 3 Lecturer in Dental Prothesis, Faculty of Dentistry, Complutense University of Madrid, Spain; 4 Senior Lecturer in Oral Surgery, Faculty of Dentistry, Complutense University of Madrid, Spain

## Abstract

**Background:**

The main clinical application of electromyography is to detect abnormalities in muscle function, to assess muscle activity for purposes of recruitment, and in the biomechanics of movement.

**Objectives:**

To analyze electromyography (EMG) findings for masticatory muscles during chewing following surgical extraction of lower third molars, and to determine any correlation between pain, inflammation, trismus, and the EMG data registered.

**Material and Methods:**

This prospective study included 31 patients. Surface EMG was used to study masseter and temporalis muscle function before lower third molar extraction and 72 hours and seven days after surgery. Clinical variables, pain, inflammation, and trismus were registered before and after surgery.

**Results:**

Studying the area and size of the masticatory muscles, higher values were found for temporalis than masseter muscles, regardless of the surgical side, which points to the greater involvement of the temporalis muscle in mastication. Comparing the side where surgery had been performed with the non-surgical side, a sharp and statistically significant reduction in amplitude and area were noted on the surgical side reflecting major functional affectation. One week after surgery, amplitude and area had almost returned to base-line values, indicating almost complete recovery. While pain decreased progressively after surgery, inflammation peaked at 72 hours, while mouth opening reached a minimum at this time, returning to normality within the week.

**Conclusions:**

Surgical extraction of lower third molars produces changes to electromyography activity that are more evident during the first hours after surgery and closely related to the intensity of pain suffered and the patient’s inflammatory responses, although they are not related to mouth opening capacity.

** Key words:**Third molar surgery, electromyography, pain, inflammation, trismus, masticatory muscles.

## Introduction

Third molar extraction is the most common type of maxillofacial and oral surgery. Impacted third molars, and their attempts to erupt, trigger a variety of pathological conditions in some 75% of cases, calling for their surgical extraction (1).

Complications related to third molar extraction have been well documented. Major complications following extraction are defined as those requiring treatment, which sometimes causes irreversible damage. Among these are abscesses, cases of uncontrollable bleeding, neurological damage, and mandibular fracture (2). Minor complications are defined as those that resolve spontaneously without treatment: pain, trismus, and inflammation. Minor complications are more frequent than major complications but the methods used to evaluate them are sometimes not very objective, particularly with regard to analyzing masticatory muscle function (3).

Following third molar extraction, many patients report restricted mouth opening, in other words, trismus. Many methods are used to assess patient discomfort and mouth opening capacity after surgery, such as visual analogue scales, quality of life questionnaires, and measurement of inter-incisal distance. But these techniques show little objectivity and do not determine muscle function (4).

Electromyography (EMG) is a technique that allows the registration of electrical activity generated by muscle cells when these are stimulated electrically or neurologically. The signals emitted are measured on the skin surface as the sum of electrical activity of muscle fiber bundles (5,6).

The main clinical application of EMG is to detect abnormalities in muscle function, to assess muscle activity for purposes of recruitment, and in the biomechanics of movement. In dentistry, EMG has mainly been used for analyzing masticatory function, but few studies have evaluated masticatory muscle activity after third molar extraction and any correlation between EMG muscle activity and the minor complications derived from surgery (7).

The objective of this study was to evaluate EMG activity of the masticatory/mouth closing muscles after third molar extraction surgery and to correlate the findings with the presence of minor complications. This prospective study performed baseline EMG before third molar extraction surgery, and at 72 hours and 7 days after surgery, measuring temporalis and masseter muscle activity with surface electrodes. Pain, inflammation and trismus parameters were also evaluated in order to identify any correlation between these and the EMG parameters registered.

## Material and Methods

-Study design 

This is a longitudinal prospective study approved on December 5, 2015, by the Institutional Ethical Committee of the Complutense University of Madrid, under protocol number CEIC-09/2015. Free and informed consent was obteined from participants in writing prior to conducting the research. The recommendations from Strengthening the Reporting of Observacional Studies in Epidemiology (STROBE) were followed for the study´s design and development (18).

A patient sample was selected according to pre-established criteria; patients underwent an EMG study before and after surgical extraction of impacted lower third molars, in order to evaluate mouth closing muscle function while chewing following an evaluation chronogram: baseline, 72 hours after extraction surgery, and 7 days after surgery.

-Patients

The study included 38 patients (25 women and 13 men with a mean age of 22.26 years), who attended the Department of Medicine and oral surgery, Faculty of Medicine and Dentistry, the Complutense University of Madrid (Spain) from January to June 2016; EMG was performed at the Department’s EMG Unit.

Of the initial 38 patients, seven were excluded as they had taken analgesics during the study period. Before the study began, each patient gave his/her informed consent to undergo third molar extraction, and to take part in the EMG study. The dentist, who performed all clinical procedures, recorded the patients’ clinical histories and performed a radiographic study from orthopantomographs, a usual procedure in this type of surgery.

Inclusion criteria were: patients classified as ASA I or II according to the American Society of Anesthesiologists classification (19), patients with mesioangular impacted third molars in the right hemi-mandible, presenting II/B position and depth according to Pell and Gregorys’ classification (20), requiring incision, mucoperiosteal flap elevation, and osteotomy.

Exclusion criteria were: patients presenting temporomandibular disorders, patients with multiple missing teeth, facial asymmetries, or patients classified as ASA III or IV. Patients who requested oral analgesic administration during the post-operative period were excluded, as only local measures were applied: chlorhexidine mouthwash and a soft cold food diet.

-Study variables

The study’s predictive variable was muscle function registered during chewing before and after lower third molar extraction surgery. The outcome variables evaluated were the amplitude and area of the masseter and temporalis muscles. The term muscle amplitude refers to muscular contraction, so that the greater the amplitude, the greater the contraction. The term area refers to the muscle’s attempts to recruit more motor units and so achieve a correct function. Other variables registered included patient age and gender, post-operative pain, inflammation, and mouth opening/trismus.

-Electromyography (EMG)

EMG was performed to evaluate muscular function on the surgical and collateral sides during chewing (the food masticated was fried potatoes) following the following evaluation chronogram: pre-operative baseline EMG, 72 hours after surgery, and 7 days after surgery. EMG was performed with surface electrodes placed on the patient’s skin over the masseter and temporalis muscles to quantify the amplitude and area in ?v over a period of 250 ms. Two surface electrodes were applied to each muscle in order to register the difference in voltage between the two independent electrodes.

-Lower third molar extraction surgery 

In all cases, surgery was performed by the same surgeon, following the same procedure under local anesthesia blocking the inferior alveolar nerve, lingual nerve, and afterwards the buccal nerve. A scalloped incision was made from the first molar with distal release directed posteriorly towards the ramus, followed by mucoperiosteal detachment, osteotomy using a handpiece with micromotor and a round carbide tungsten bur, extraction, bone regularization, and 5-0 silk sutures. Patients received no oral drug treatment in order to avoid data bias.

-Data collection

Evaluation of inflammation and mouth opening/trismus parameters were performed at the same study times as the EMG evaluations. For pain measurement, baseline registration was not pre-surgical but rather the pain experienced on the same day as surgery was measured, and therafter at 72 hours and one week after surgery. Pain was evaluated using a visual analogue scale (VAS), whereby the patient scored pain intensity on a scale from 0 to 10, 0 indicating absence of pain and 10 maximum pain. Inflammation was evaluated following the method proposed by Amin and Laskin (21), before surgery, 72 hours after surgery, and one week after surgery, registering the distances from the tragus to the oral commissure, from the tragus to the symphysis, and from the outer corner of the eye to the gonial angle, by placing a silk thread secured by mosquito forceps, that was later measured with a millimeter ruler. Mouth opening/trismus was measured using a micrometer caliper, which measures intercisal distance at maximum opening in millimeters. Mouth opening was measured just before surgery, and 72 hours, and one week after surgery.

-Statistical data analysis

Data were entered in a Microsoft Excel spreadsheet (Microsoft, Redmond WA, USA) and processed using SPSS software (version 21.0; SPSS, IBM, Chicago, IL, USA). Pearson’s test was used to study correlations between two means and ANOVA F for more than two means, compared pairwise by the Bonferoni test.

## Results

-Descriptive study of variables analyzed 

Mean patient age was 22.26 ± 2.59. The ratio of men/women was 2.5/0.6.

**Figure 1:**
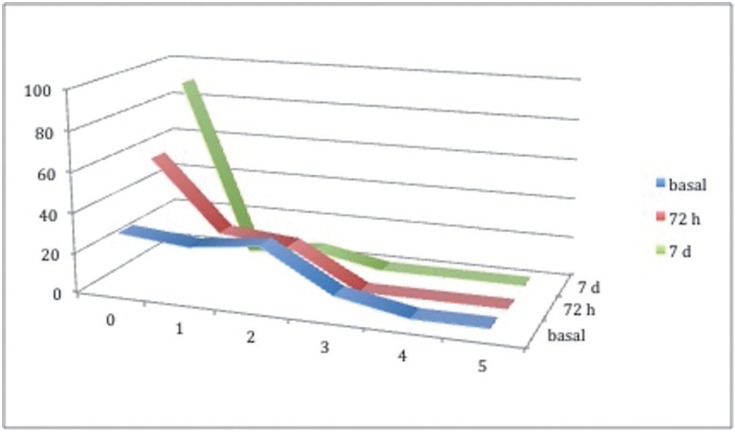
Percentage of patients according to pain classification on a scale of 0-10 at the three study times.

The pain suffered by patients registered by VAS was found to diminish gradually and progressively from each study time to the next. It should be noted that over 90% of the patients did not experience any pain at the 7-day mark. In the initial evaluation following surgery, patients reported pain levels 4 and 5 in 3.2% cases, level 3 in 9.8%, and levels 2,1 and 0 in 29%, 25.8% and 29% respectively. At 72 hours, the number of patients not experiencing any pain increased substantially to 58.1%, with cases reporting level 1, representing 22.6%, and level 2 19.4%; no patient reported pain intensity at higher levels. The final evaluation at 7 days made it clear that the majority of patients (90.3% of the sample) felt no pain by this time; one patient (3.2%) reported level 1 pain and two patients (6.5%) level 2 (Fig. 1).

**Figure 2:**
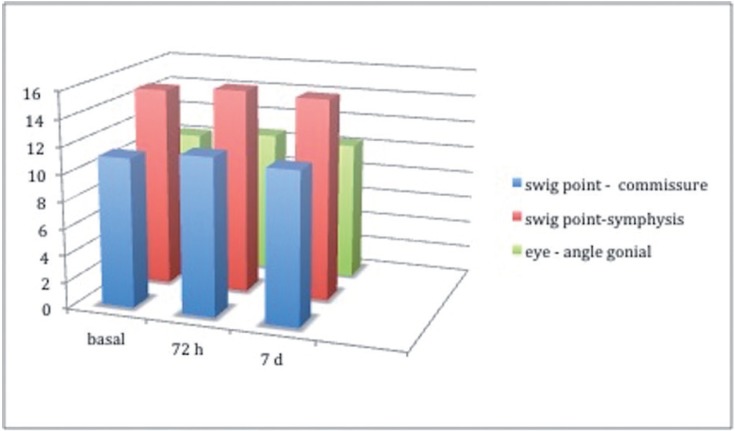
Comparison of inflammation at the three study times. Three distances were measured in centimeters.

Evaluations of inflammation showed a slight increase in mean value at 72 hours in comparison with the base-line evaluation, and thereafter showed a progressive decrease until almost returning to the mean baseline value. At baseline, the mean tragus-oral commissure value was 11.14 cm, tragus-symphysis 14.94, and outer corner of the eye-gonial angle was 10.20cm. Mean values registered at 7 days were: tragus-oral commissure, 11,33 cm; tragus-symphysis, 15,05cm; and outer corner of the eye-gonial angle, 10.35 cm (Fig. 2).

**Figure 3:**
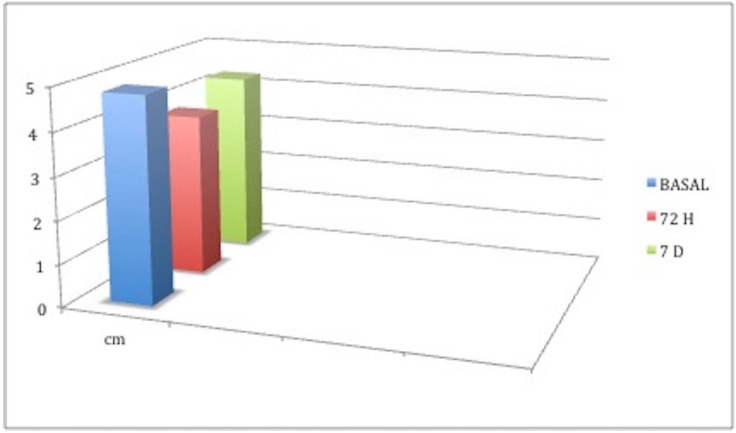
Comparison of mouth opening at the three study times. Intercisal distances were measured in centimeters.

Likewise, mean mouth opening at baseline was 48.1 mm, deceasing to a mean value of 38.16mm at 72 hours, and 42.77mm at 7 days (Fig. 3)

-Distribution of EMG data for masseter and temporalis muscles measured with surface electrodes

Analyzing EMG values registered by surface electrodes placed over masseter and temporalis muscles during chewing at each study time, amplitude and area were greater in the temporalis than masseter muscles on both sides at all study times. Between baseline and 72 hours, a clear decrease was registered in the area and amplitude for both muscles, which was greater on the surgical side, but without statistically significant difference between sides.

After 7 days, recovery of the masseter and temporalis muscles on the non-surgical side was significantly greater than the surgical side (masseter amplitude, *p*=0.025; masseter area, *p*=N.S; temporalis amplitude, *p*=0.051; temporalis area, *p*<0.001), but a clear tendency for both muscles to recover was observed on both sides ( Table 1).

**Table 1:**
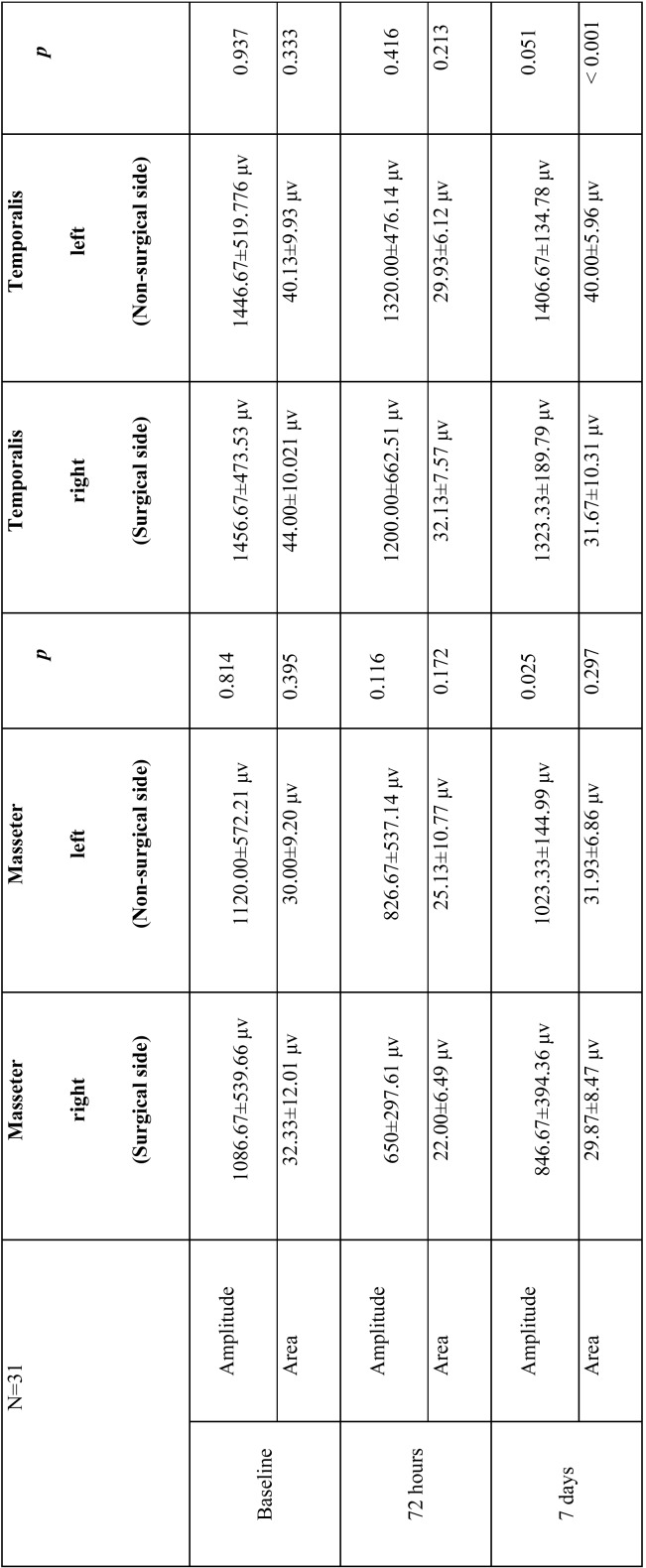
Amplitude and area of masseter and temporalis muscles at the three study times.

-Analysis of correlations between electromyography results and other variables

Statistical analysis identified some significant correlations between the study variables. Regarding age, younger patients recovered more slowly than older patients (Pearson *p*=0.37). No correlation was found between patient age and pain or inflammation. As for gender, women (mean=1.64) reported greater pain than men (mean=0.5), with statistically significant difference at the first evaluation time on the day of surgery (*p*=0.049), but without significant difference at 72 hours or seven days after surgery. No correlation was found between patient gender and intercisal distance at any of the study times. However, inflammation was significantly higher in men than women at all three study times ( Table 2).

**Table 2:**
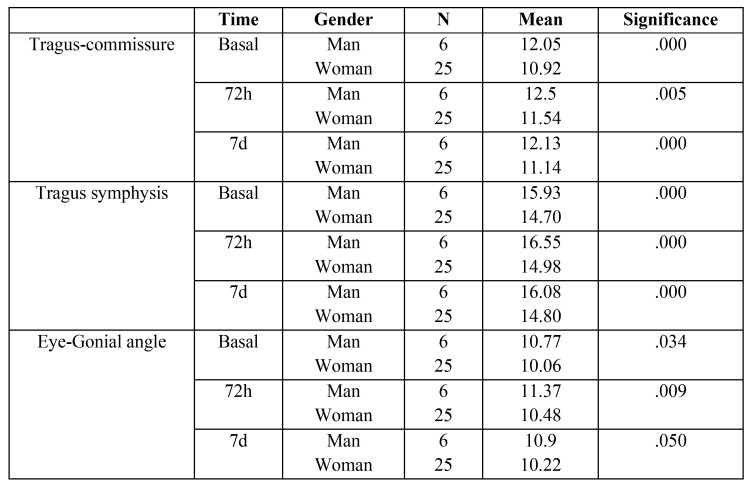
Correlation between patient gender and inflammation at the three study times.

A statistically significant correlation was found between pain and muscle amplitude and area reduction at the first two study times. Seven days after surgery, there was no significant correlation, as pain had mostly disappeared and muscular function had been restored, presenting similar amplitude and area values to baseline.

Following the same pattern, after 72 hours a positive correlation was found between the degree of inflammation and muscle amplitude and area; the greater the inflammation, the less the muscle amplitude and area. After 7 days, muscle amplitude and area values had been re-established in accord with the reduction in inflammation, indicating the recovery of muscle function.

No statistically significant results were registered to correlate muscle function during chewing with changes to intercisal distance, from which it may be concluded that functional disorders of the masseter and temporalis muscles do not determine mouth-opening capacity.

## Discussion

The literature includes numerous articles on the signs and symptoms arising from third molar impaction, indications for its extraction, and post-operative complications deriving from extraction, which together have generated some controversy. There is a wide range of opinion as to the frequency, order of appearance and duration of impacted third molars, but all researchers agree on a trio of symptoms that derive from extraction: pain, trismus, and inflammation. The value of the present study lies in its EMG evaluation of masticatory muscle behavior in relation to pain, mouth opening/trismus, and inflammation.

Complications related to the masticatory muscles, such as trismus and inflammation are subjective parameters, and so difficult to quantify objectively (8). Electromyography data make it possible to measure the duration of muscle affectation, muscle action potentials (9), and motor unit firing frequency (10).

Motor units contribute to signals passed to surface EMG, which are, as the name suggests, on the surface. Any decrease in EMG activity is due to a reduction in surface muscle fiber activity (11). But the masseter muscle adjacent to a third molar is situated at a deeper level. Therefore it is possible that direct masseter muscle damage after surgery remains undetected by surface EMG. Nevertheless, the temporalis muscle fibers adjacent to a third molar reach the surface, which explains why at all study times amplitude and area values for this muscle were higher than those registered for the masseter muscles.

EMG data for amplitude and area of masseter and temporalis muscles showed significantly different values between the surgical and non-surgical sides 7 days after surgery, with lower values on the surgical side than the non-surgical side. But some researchers such as Moraes MB *et al.* (17), who conducted a study of 20 patients undergoing mandibular lower third molar extractions, did not find changes to muscle tone and found no significant differences between left and right temporal and masseter muscles. In the present study, EMG activity of the masticatory muscles on the surgical side was clearly seen to diminish during chewing, as reflected in the results of other research (12). The recovery of EMG activity could be due to functional adaptation of muscle coordination, facilitating these muscles’ movement (13); if this is the case, the higher amplitude and area values registered on the non-surgical side could be due to functional adaptation produced after third molar extraction.

The present results show a positive correlation between the presence of pain and a reduction in electromyography activity in the masticatory muscles with pain subsiding by the 7-day study time, when the correlation was reversed. And so it may be concluded – as other authors have done – that pain experienced on the extraction side leads to a reduction in muscle activity as a defense mechanism; the reduced activity, and therefore function, is considered an innate analgesic mechanism exercised to avoid pain (14).

The possible correlation between post-operative inflammation and EMG activity in the masseter and temporalis muscles was also investigated. Inflammation peaked at 72 hours, coinciding with a significant decrease in amplitude and area. The release of inflammation mediators causes pain and oedema, which limit jaw movements (15). In addition, acute inflammation derived from surgery may result in spasms of the mouth closing muscles, which will lead to limited mouth opening (16). The present study did not find a statistically significant correlation between reduced mouth opening and EMG data, although the lowest mouth opening values were registered at 72 hours, coinciding with maximum inflammation and therefore the lowest muscular amplitude and area values.

To sum up, lower third molar extraction alters post-operative EMG variables in the mouth closing/masticatory muscles. Seven days after surgery, statistically significant differences were found between amplitude and area values in favor of the non-surgical side, which points to greater functional recovery of the muscles on the antagonist side. The EMG alterations observed would appear to be transitory as there was a significant improvement in amplitude and area values from the 72-hour to the 7-day study time, regardless of the side intervened. Although the reliability of EMG readings has been a topic of some controversy, it may be considered an objective method for evaluating muscle function. This method can be used to assess reduced masticatory function of the masseter and temporalis muscles after third molar surgery, as well as the trio of symptoms correlated to these variables – pain, inflammation, and trismus.

As in many studies, the findings were subject to a possibility of bias. One potential source of bias is the fact that the capacity of surface electromyography may be influenced by the individual patient’s skin type and surface grease. This is a factor that is difficult to homogenize although, according to the authors’ criteria, the phenotypic characteristics of the study population were very similar.

## Conclusions

Surgical extraction of lower third molars produces changes to electromyography activity that are more evident during the first hours after surgery and closely related to the intensity of pain suffered and the patient’s inflammatory responses, although they are not related to mouth opening capacity.
